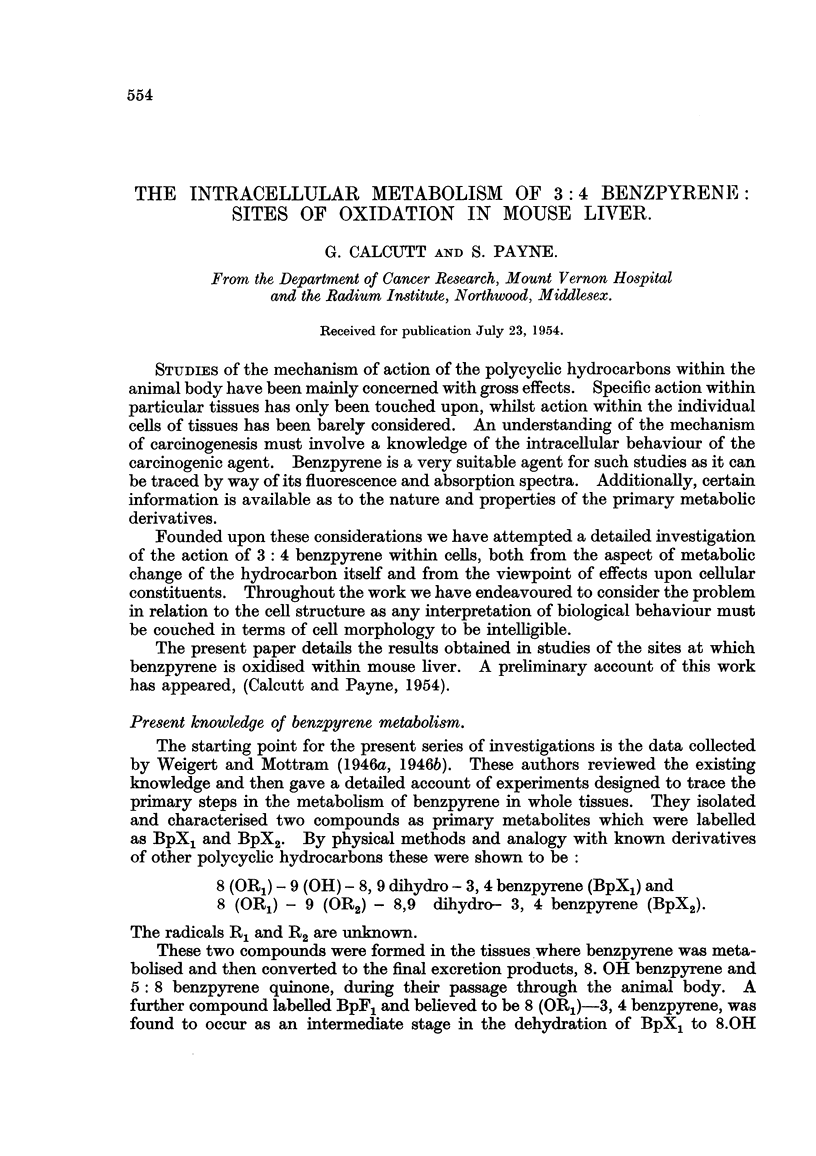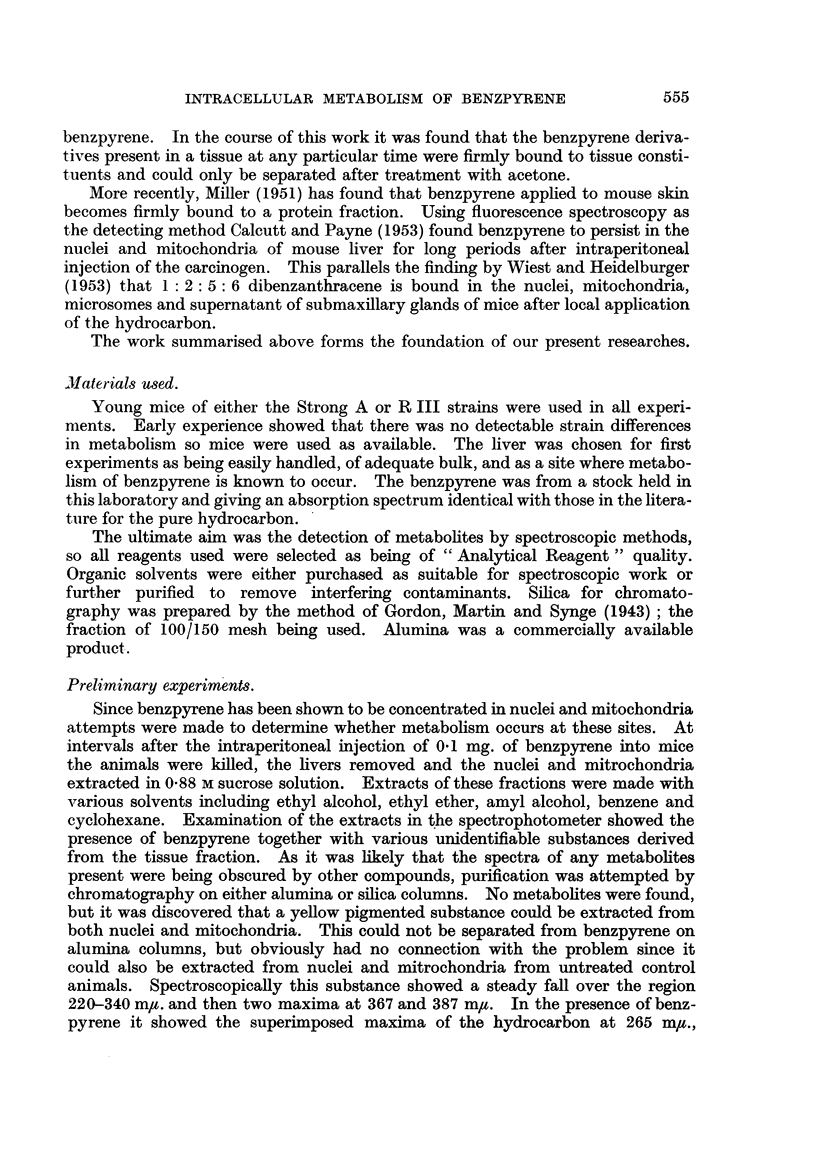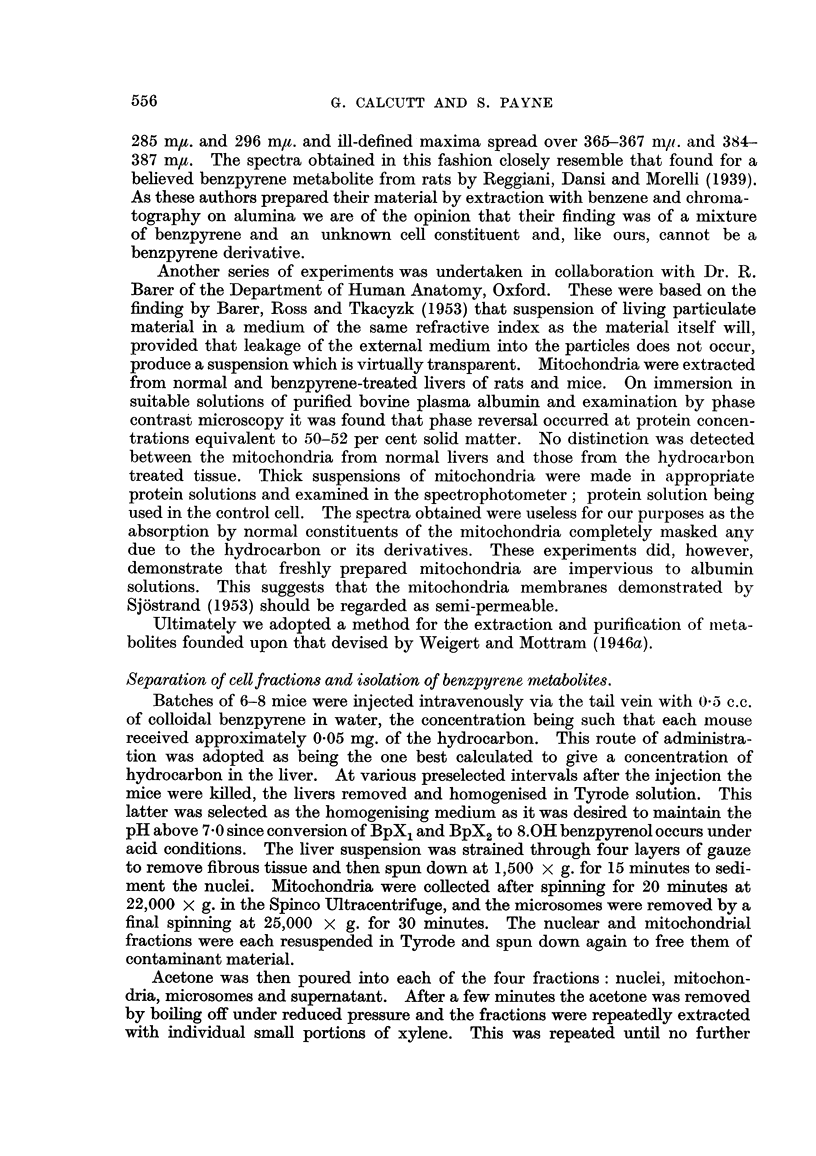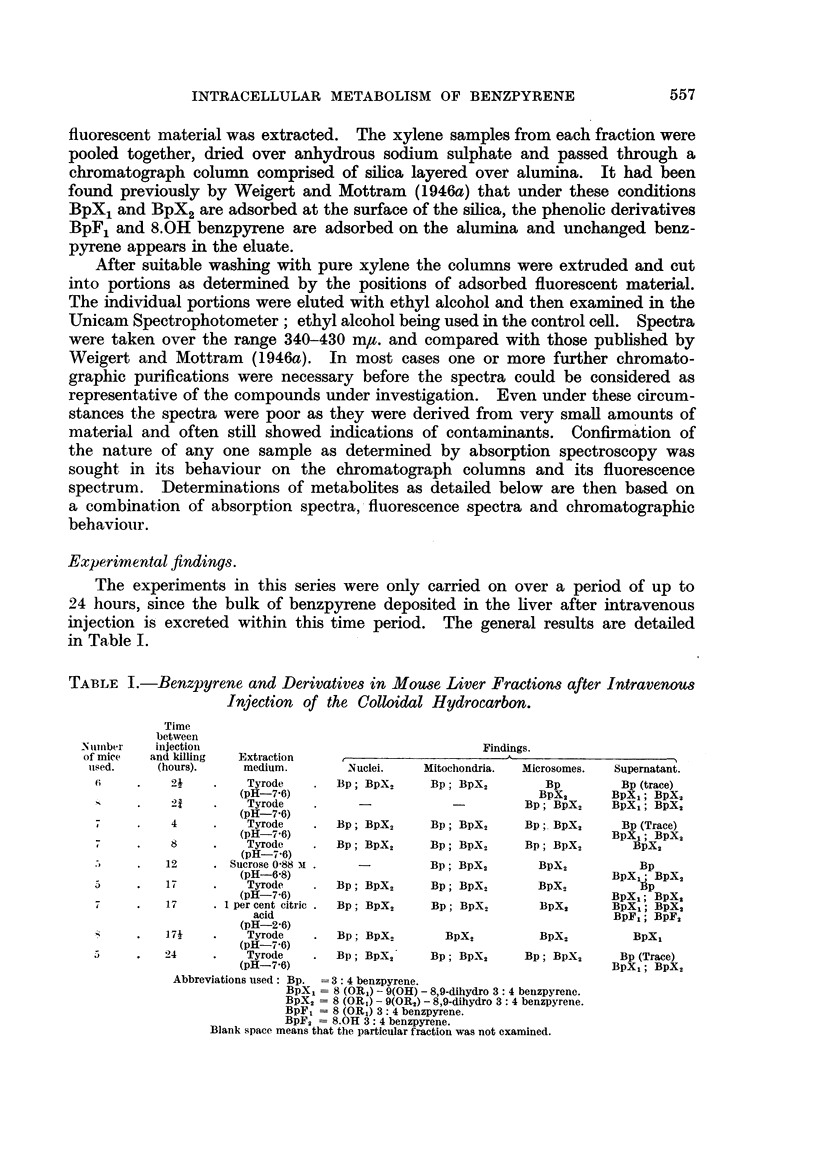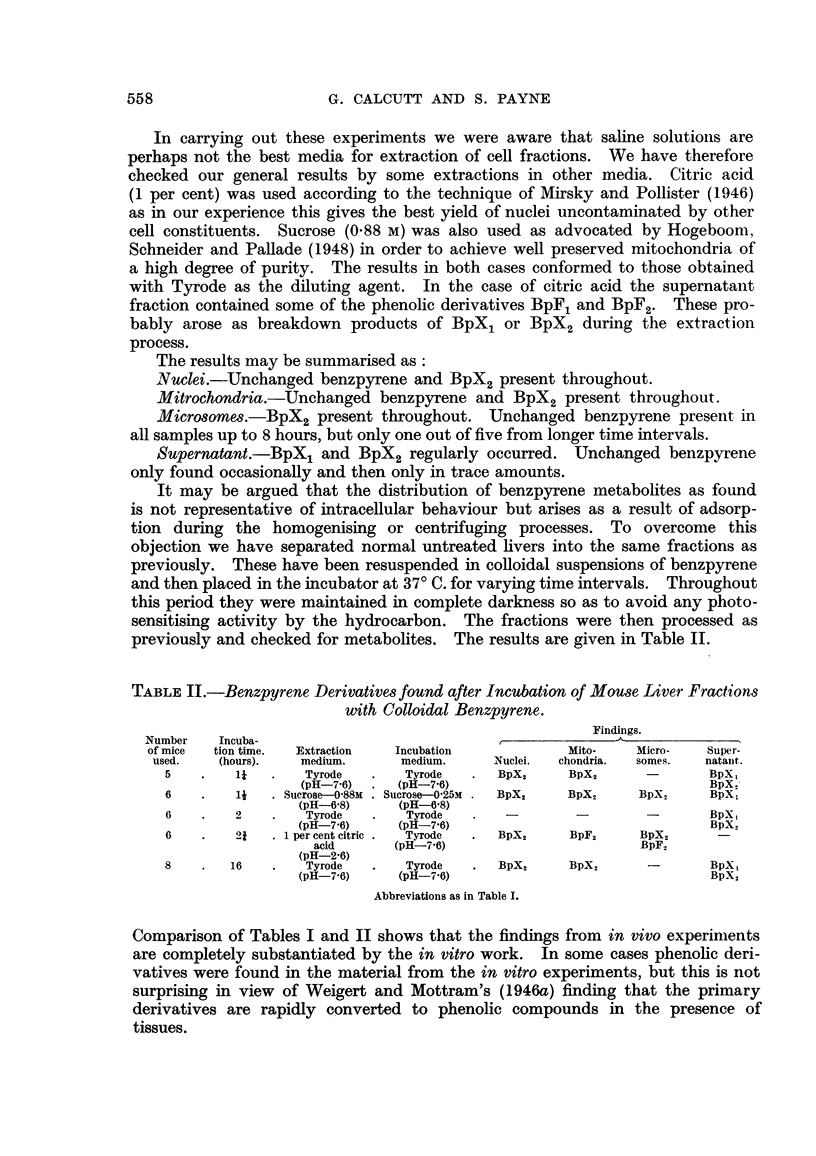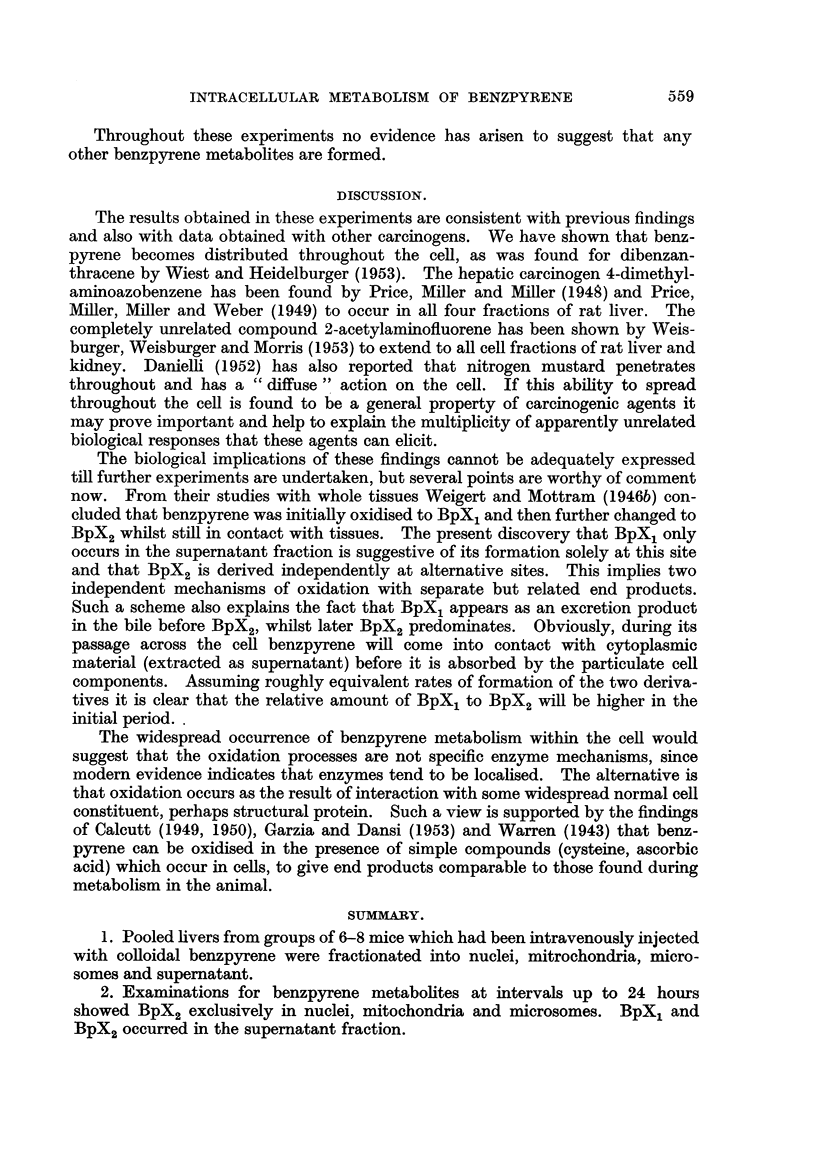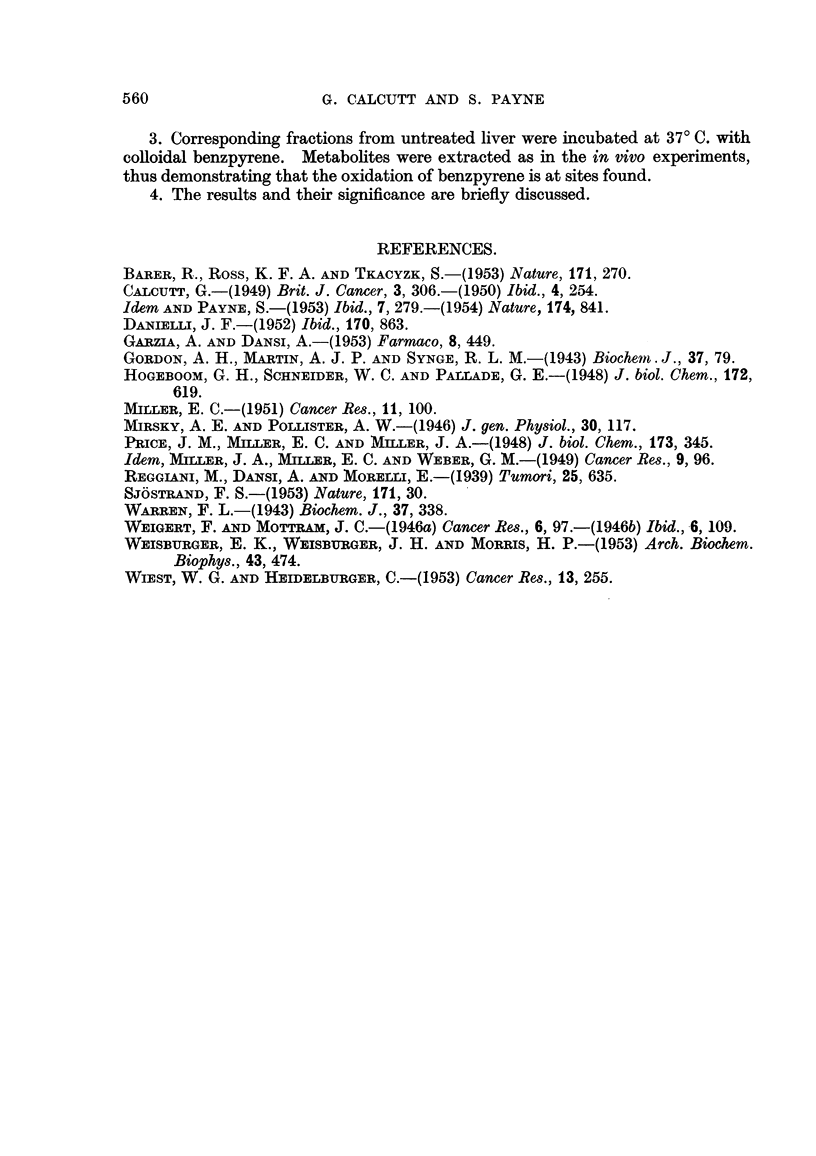# The Intracellular Metabolism of 3: 4 Benzypyrene: Sites of Oxidation in Mouse Liver

**DOI:** 10.1038/bjc.1954.59

**Published:** 1954-09

**Authors:** G. Calcutt, S. Payne


### THE INTRACELLULAR METABOLISM OF 3 : 4 BENZPYRENE: SITES OF OXIDATION IN MOUSE LIVER.

G. CALCUTT AND S. PAYNE.

*From the Department of Cancer Research, Mount Vernon Hospital and the Radium Institute, Northwood, Middlesex.*

Received for publication July 23, 1954.

STUDIES of the mechanism of action of the polycyclic hydrocarbons within the animal body have been mainly concerned with gross effects. Specific action within particular tissues has only been touched upon, whilst action within the individual cells of tissues has been barely considered. An understanding of the mechanism of carcinogenesis must involve a knowledge of the intracellular behaviour of the carcinogenic agent. Benzpyrene is a very suitable agent for such studies as it can be traced by way of its fluorescence and absorption spectra. Additionally, certain information is available as to the nature and properties of the primary metabolic derivatives.

Founded upon these considerations we have attempted a detailed investigation of the action of 3 : 4 benzpyrene within cells, both from the aspect of metabolic change of the hydrocarbon itself and from the viewpoint of effects upon cellular constituents. Throughout the work we have endeavoured to consider the problem in relation to the cell structure as any interpretation of biological behaviour must be couched in terms of cell morphology to be intelligible.

The present paper details the results obtained in studies of the sites at which benzpyrene is oxidised within mouse liver. A preliminary account of this work has appeared, (Calcutt and Payne, 1954).

*Present knowledge of benzpyrene metabolism.*

The starting point for the present series of investigations is the data collected by Weigert and Mottram (1946*a*, 1946*b*). These authors reviewed the existing knowledge and then gave a detailed account of experiments designed to trace the primary steps in the metabolism of benzpyrene in whole tissues. They isolated and characterised two compounds as primary metabolites which were labelled as $BpX_1$ and $BpX_2$. By physical methods and analogy with known derivatives of other polycyclic hydrocarbons these were shown to be :

8 $(OR_1)$ – 9 (OH) – 8, 9 dihydro – 3, 4 benzpyrene $(BpX_1)$ and
8 $(OR_1)$ – 9 $(OR_2)$ – 8,9 dihydro– 3, 4 benzpyrene $(BpX_2)$.

The radicals $R_1$ and $R_2$ are unknown.

These two compounds were formed in the tissues where benzpyrene was metabolised and then converted to the final excretion products, 8. OH benzpyrene and 5 : 8 benzpyrene quinone, during their passage through the animal body. A further compound labelled $BpF_1$ and believed to be 8 $(OR_1)$—3, 4 benzpyrene, was found to occur as an intermediate stage in the dehydration of $BpX_1$ to 8.OH

benzpyrene. In the course of this work it was found that the benzpyrene derivatives present in a tissue at any particular time were firmly bound to tissue constituents and could only be separated after treatment with acetone.

More recently, Miller (1951) has found that benzpyrene applied to mouse skin becomes firmly bound to a protein fraction. Using fluorescence spectroscopy as the detecting method Calcutt and Payne (1953) found benzpyrene to persist in the nuclei and mitochondria of mouse liver for long periods after intraperitoneal injection of the carcinogen. This parallels the finding by Wiest and Heidelburger (1953) that 1 : 2 : 5 : 6 dibenzanthracene is bound in the nuclei, mitochondria, microsomes and supernatant of submaxillary glands of mice after local application of the hydrocarbon.

The work summarised above forms the foundation of our present researches.

*Materials used.*

Young mice of either the Strong A or R III strains were used in all experiments. Early experience showed that there was no detectable strain differences in metabolism so mice were used as available. The liver was chosen for first experiments as being easily handled, of adequate bulk, and as a site where metabolism of benzpyrene is known to occur. The benzpyrene was from a stock held in this laboratory and giving an absorption spectrum identical with those in the literature for the pure hydrocarbon.

The ultimate aim was the detection of metabolites by spectroscopic methods, so all reagents used were selected as being of " Analytical Reagent " quality. Organic solvents were either purchased as suitable for spectroscopic work or further purified to remove interfering contaminants. Silica for chromatography was prepared by the method of Gordon, Martin and Synge (1943) ; the fraction of 100/150 mesh being used. Alumina was a commercially available product.

*Preliminary experiments.*

Since benzpyrene has been shown to be concentrated in nuclei and mitochondria attempts were made to determine whether metabolism occurs at these sites. At intervals after the intraperitoneal injection of 0·1 mg. of benzpyrene into mice the animals were killed, the livers removed and the nuclei and mitrochondria extracted in 0·88 M sucrose solution. Extracts of these fractions were made with various solvents including ethyl alcohol, ethyl ether, amyl alcohol, benzene and cyclohexane. Examination of the extracts in the spectrophotometer showed the presence of benzpyrene together with various unidentifiable substances derived from the tissue fraction. As it was likely that the spectra of any metabolites present were being obscured by other compounds, purification was attempted by chromatography on either alumina or silica columns. No metabolites were found, but it was discovered that a yellow pigmented substance could be extracted from both nuclei and mitochondria. This could not be separated from benzpyrene on alumina columns, but obviously had no connection with the problem since it could also be extracted from nuclei and mitrochondria from untreated control animals. Spectroscopically this substance showed a steady fall over the region 220–340 m$\mu$. and then two maxima at 367 and 387 m$\mu$. In the presence of benzpyrene it showed the superimposed maxima of the hydrocarbon at 265 m$\mu$.,

285 mμ. and 296 mμ. and ill-defined maxima spread over 365–367 mμ. and 384–387 mμ. The spectra obtained in this fashion closely resemble that found for a believed benzpyrene metabolite from rats by Reggiani, Dansi and Morelli (1939). As these authors prepared their material by extraction with benzene and chromatography on alumina we are of the opinion that their finding was of a mixture of benzpyrene and an unknown cell constituent and, like ours, cannot be a benzpyrene derivative.

Another series of experiments was undertaken in collaboration with Dr. R. Barer of the Department of Human Anatomy, Oxford. These were based on the finding by Barer, Ross and Tkacyzk (1953) that suspension of living particulate material in a medium of the same refractive index as the material itself will, provided that leakage of the external medium into the particles does not occur, produce a suspension which is virtually transparent. Mitochondria were extracted from normal and benzpyrene-treated livers of rats and mice. On immersion in suitable solutions of purified bovine plasma albumin and examination by phase contrast microscopy it was found that phase reversal occurred at protein concentrations equivalent to 50–52 per cent solid matter. No distinction was detected between the mitochondria from normal livers and those from the hydrocarbon treated tissue. Thick suspensions of mitochondria were made in appropriate protein solutions and examined in the spectrophotometer ; protein solution being used in the control cell. The spectra obtained were useless for our purposes as the absorption by normal constituents of the mitochondria completely masked any due to the hydrocarbon or its derivatives. These experiments did, however, demonstrate that freshly prepared mitochondria are impervious to albumin solutions. This suggests that the mitochondria membranes demonstrated by Sjöstrand (1953) should be regarded as semi-permeable.

Ultimately we adopted a method for the extraction and purification of metabolites founded upon that devised by Weigert and Mottram (1946*a*).

*Separation of cell fractions and isolation of benzpyrene metabolites.*

Batches of 6–8 mice were injected intravenously via the tail vein with 0·5 c.c. of colloidal benzpyrene in water, the concentration being such that each mouse received approximately 0·05 mg. of the hydrocarbon. This route of administration was adopted as being the one best calculated to give a concentration of hydrocarbon in the liver. At various preselected intervals after the injection the mice were killed, the livers removed and homogenised in Tyrode solution. This latter was selected as the homogenising medium as it was desired to maintain the pH above 7·0 since conversion of $BpX_1$ and $BpX_2$ to 8.OH benzpyrenol occurs under acid conditions. The liver suspension was strained through four layers of gauze to remove fibrous tissue and then spun down at 1,500 × g. for 15 minutes to sediment the nuclei. Mitochondria were collected after spinning for 20 minutes at 22,000 × g. in the Spinco Ultracentrifuge, and the microsomes were removed by a final spinning at 25,000 × g. for 30 minutes. The nuclear and mitochondrial fractions were each resuspended in Tyrode and spun down again to free them of contaminant material.

Acetone was then poured into each of the four fractions : nuclei, mitochondria, microsomes and supernatant. After a few minutes the acetone was removed by boiling off under reduced pressure and the fractions were repeatedly extracted with individual small portions of xylene. This was repeated until no further fluorescent material was extracted. The xylene samples from each fraction were pooled together, dried over anhydrous sodium sulphate and passed through a chromatograph column comprised of silica layered over alumina. It had been found previously by Weigert and Mottram (1946*a*) that under these conditions $BpX_1$ and $BpX_2$ are adsorbed at the surface of the silica, the phenolic derivatives $BpF_1$ and 8.OH benzpyrene are adsorbed on the alumina and unchanged benzpyrene appears in the eluate.

After suitable washing with pure xylene the columns were extruded and cut into portions as determined by the positions of adsorbed fluorescent material. The individual portions were eluted with ethyl alcohol and then examined in the Unicam Spectrophotometer ; ethyl alcohol being used in the control cell. Spectra were taken over the range 340–430 m$\mu$. and compared with those published by Weigert and Mottram (1946*a*). In most cases one or more further chromatographic purifications were necessary before the spectra could be considered as representative of the compounds under investigation. Even under these circumstances the spectra were poor as they were derived from very small amounts of material and often still showed indications of contaminants. Confirmation of the nature of any one sample as determined by absorption spectroscopy was sought in its behaviour on the chromatograph columns and its fluorescence spectrum. Determinations of metabolites as detailed below are then based on a combination of absorption spectra, fluorescence spectra and chromatographic behaviour.

*Experimental findings.*

The experiments in this series were only carried on over a period of up to 24 hours, since the bulk of benzpyrene deposited in the liver after intravenous injection is excreted within this time period. The general results are detailed in Table I.

TABLE I.—*Benzpyrene and Derivatives in Mouse Liver Fractions after Intravenous Injection of the Colloidal Hydrocarbon.*

| Number of mice used. | Time between injection and killing (hours). | Extraction medium. | Findings. | | | |
|---|---|---|---|---|---|---|
| | | | Nuclei. | Mitochondria. | Microsomes. | Supernatant. |
| 6 | $2\frac{1}{2}$ | Tyrode (pH—7·6) | Bp ; $BpX_2$ | Bp ; $BpX_2$ | Bp $BpX_2$ | Bp (trace) $BpX_1$ ; $BpX_2$ |
| 8 | $2\frac{3}{4}$ | Tyrode (pH—7·6) | — | — | Bp ; $BpX_2$ | $BpX_1$ ; $BpX_2$ |
| 7 | 4 | Tyrode (pH—7·6) | Bp ; $BpX_2$ | Bp ; $BpX_2$ | Bp ; $BpX_2$ | Bp (Trace) $BpX_1$ ; $BpX_2$ |
| 7 | 8 | Tyrode (pH—7·6) | Bp ; $BpX_2$ | Bp ; $BpX_2$ | Bp ; $BpX_2$ | $BpX_2$ |
| 5 | 12 | Sucrose 0·88 M (pH—6·8) | — | Bp ; $BpX_2$ | $BpX_2$ | Bp $BpX_1$ ; $BpX_2$ |
| 5 | 17 | Tyrode (pH—7·6) | Bp ; $BpX_2$ | Bp ; $BpX_2$ | $BpX_2$ | Bp $BpX_1$ ; $BpX_2$ |
| 7 | 17 | 1 per cent citric acid (pH—2·6) | Bp ; $BpX_2$ | Bp ; $BpX_2$ | $BpX_2$ | $BpX_1$ ; $BpX_2$ $BpF_1$ ; $BpF_2$ |
| 8 | $17\frac{1}{2}$ | Tyrode (pH—7·6) | Bp ; $BpX_2$ | $BpX_2$ | $BpX_2$ | $BpX_1$ |
| 5 | 24 | Tyrode (pH—7·6) | Bp ; $BpX_2$ | Bp ; $BpX_2$ | Bp ; $BpX_2$ | Bp (Trace) $BpX_1$ ; $BpX_2$ |

Abbreviations used : Bp. = 3 : 4 benzpyrene.
$BpX_1$ = 8 ($OR_1$) – 9(OH) – 8,9-dihydro 3 : 4 benzpyrene.
$BpX_2$ = 8 ($OR_1$) – 9($OR_2$) – 8,9-dihydro 3 : 4 benzpyrene.
$BpF_1$ = 8 ($OR_1$) 3 : 4 benzpyrene.
$BpF_2$ = 8.OH 3 : 4 benzpyrene.
Blank space means that the particular fraction was not examined.

In carrying out these experiments we were aware that saline solutions are perhaps not the best media for extraction of cell fractions. We have therefore checked our general results by some extractions in other media. Citric acid (1 per cent) was used according to the technique of Mirsky and Pollister (1946) as in our experience this gives the best yield of nuclei uncontaminated by other cell constituents. Sucrose (0·88 M) was also used as advocated by Hogeboom, Schneider and Pallade (1948) in order to achieve well preserved mitochondria of a high degree of purity. The results in both cases conformed to those obtained with Tyrode as the diluting agent. In the case of citric acid the supernatant fraction contained some of the phenolic derivatives $BpF_1$ and $BpF_2$. These probably arose as breakdown products of $BpX_1$ or $BpX_2$ during the extraction process.

The results may be summarised as :

*Nuclei.*—Unchanged benzpyrene and $BpX_2$ present throughout.

*Mitrochondria.*—Unchanged benzpyrene and $BpX_2$ present throughout.

*Microsomes.*—$BpX_2$ present throughout. Unchanged benzpyrene present in all samples up to 8 hours, but only one out of five from longer time intervals.

*Supernatant.*—$BpX_1$ and $BpX_2$ regularly occurred. Unchanged benzpyrene only found occasionally and then only in trace amounts.

It may be argued that the distribution of benzpyrene metabolites as found is not representative of intracellular behaviour but arises as a result of adsorption during the homogenising or centrifuging processes. To overcome this objection we have separated normal untreated livers into the same fractions as previously. These have been resuspended in colloidal suspensions of benzpyrene and then placed in the incubator at 37° C. for varying time intervals. Throughout this period they were maintained in complete darkness so as to avoid any photosensitising activity by the hydrocarbon. The fractions were then processed as previously and checked for metabolites. The results are given in Table II.

TABLE II.—*Benzpyrene Derivatives found after Incubation of Mouse Liver Fractions with Colloidal Benzpyrene.*

| Number of mice used. | Incubation time. (hours). | Extraction medium. | Incubation medium. | Findings. Nuclei. | Mitochondria. | Microsomes. | Supernatant. |
|---|---|---|---|---|---|---|---|
| 5 | 1¼ | Tyrode (pH—7·6) | Tyrode (pH—7·6) | $BpX_2$ | $BpX_2$ | — | $BpX_1$ $BpX_2$ |
| 6 | 1½ | Sucrose—0·88M (pH—6·8) | Sucrose—0·25M (pH—6·8) | $BpX_2$ | $BpX_2$ | $BpX_2$ | $BpX_1$ |
| 6 | 2 | Tyrode (pH—7·6) | Tyrode (pH—7·6) | — | — | — | $BpX_1$ $BpX_2$ |
| 6 | 2¾ | 1 per cent citric acid (pH—2·6) | Tyrode (pH—7·6) | $BpX_2$ | $BpF_2$ | $BpX_2$ $BpF_2$ | — |
| 8 | 16 | Tyrode (pH—7·6) | Tyrode (pH—7·6) | $BpX_2$ | $BpX_2$ | — | $BpX_1$ $BpX_2$ |

Abbreviations as in Table I.

Comparison of Tables I and II shows that the findings from *in vivo* experiments are completely substantiated by the *in vitro* work. In some cases phenolic derivatives were found in the material from the *in vitro* experiments, but this is not surprising in view of Weigert and Mottram's (1946*a*) finding that the primary derivatives are rapidly converted to phenolic compounds in the presence of tissues.

Throughout these experiments no evidence has arisen to suggest that any other benzpyrene metabolites are formed.

### DISCUSSION.

The results obtained in these experiments are consistent with previous findings and also with data obtained with other carcinogens. We have shown that benzpyrene becomes distributed throughout the cell, as was found for dibenzanthracene by Wiest and Heidelburger (1953). The hepatic carcinogen 4-dimethylaminoazobenzene has been found by Price, Miller and Miller (1948) and Price, Miller, Miller and Weber (1949) to occur in all four fractions of rat liver. The completely unrelated compound 2-acetylaminofluorene has been shown by Weisburger, Weisburger and Morris (1953) to extend to all cell fractions of rat liver and kidney. Danielli (1952) has also reported that nitrogen mustard penetrates throughout and has a " diffuse " action on the cell. If this ability to spread throughout the cell is found to be a general property of carcinogenic agents it may prove important and help to explain the multiplicity of apparently unrelated biological responses that these agents can elicit.

The biological implications of these findings cannot be adequately expressed till further experiments are undertaken, but several points are worthy of comment now. From their studies with whole tissues Weigert and Mottram (1946*b*) concluded that benzpyrene was initially oxidised to $BpX_1$ and then further changed to $BpX_2$ whilst still in contact with tissues. The present discovery that $BpX_1$ only occurs in the supernatant fraction is suggestive of its formation solely at this site and that $BpX_2$ is derived independently at alternative sites. This implies two independent mechanisms of oxidation with separate but related end products. Such a scheme also explains the fact that $BpX_1$ appears as an excretion product in the bile before $BpX_2$, whilst later $BpX_2$ predominates. Obviously, during its passage across the cell benzpyrene will come into contact with cytoplasmic material (extracted as supernatant) before it is absorbed by the particulate cell components. Assuming roughly equivalent rates of formation of the two derivatives it is clear that the relative amount of $BpX_1$ to $BpX_2$ will be higher in the initial period. .

The widespread occurrence of benzpyrene metabolism within the cell would suggest that the oxidation processes are not specific enzyme mechanisms, since modern evidence indicates that enzymes tend to be localised. The alternative is that oxidation occurs as the result of interaction with some widespread normal cell constituent, perhaps structural protein. Such a view is supported by the findings of Calcutt (1949, 1950), Garzia and Dansi (1953) and Warren (1943) that benzpyrene can be oxidised in the presence of simple compounds (cysteine, ascorbic acid) which occur in cells, to give end products comparable to those found during metabolism in the animal.

### SUMMARY.

1. Pooled livers from groups of 6–8 mice which had been intravenously injected with colloidal benzpyrene were fractionated into nuclei, mitrochondria, microsomes and supernatant.

2. Examinations for benzpyrene metabolites at intervals up to 24 hours showed $BpX_2$ exclusively in nuclei, mitochondria and microsomes. $BpX_1$ and $BpX_2$ occurred in the supernatant fraction.

3. Corresponding fractions from untreated liver were incubated at 37° C. with colloidal benzpyrene. Metabolites were extracted as in the *in vivo* experiments, thus demonstrating that the oxidation of benzpyrene is at sites found.

4. The results and their significance are briefly discussed.

### REFERENCES.

Barer, R., Ross, K. F. A. and Tkacyzk, S.—(1953) *Nature*, **171**, 270.

Calcutt, G.—(1949) *Brit. J. Cancer*, **3**, 306.—(1950) *Ibid.*, **4**, 254.

*Idem* and Payne, S.—(1953) *Ibid.*, **7**, 279.—(1954) *Nature*, **174**, 841.

Danielli, J. F.—(1952) *Ibid.*, **170**, 863.

Garzia, A. and Dansi, A.—(1953) *Farmaco*, **8**, 449.

Gordon, A. H., Martin, A. J. P. and Synge, R. L. M.—(1943) *Biochem. J.*, **37**, 79.

Hogeboom, G. H., Schneider, W. C. and Pallade, G. E.—(1948) *J. biol. Chem.*, **172**, 619.

Miller, E. C.—(1951) *Cancer Res.*, **11**, 100.

Mirsky, A. E. and Pollister, A. W.—(1946) *J. gen. Physiol.*, **30**, 117.

Price, J. M., Miller, E. C. and Miller, J. A.—(1948) *J. biol. Chem.*, **173**, 345.

*Idem*, Miller, J. A., Miller, E. C. and Weber, G. M.—(1949) *Cancer Res.*, **9**, 96.

Reggiani, M., Dansi, A. and Morelli, E.—(1939) *Tumori*, **25**, 635.

Sjöstrand, F. S.—(1953) *Nature*, **171**, 30.

Warren, F. L.—(1943) *Biochem. J.*, **37**, 338.

Weigert, F. and Mottram, J. C.—(1946*a*) *Cancer Res.*, **6**, 97.—(1946*b*) *Ibid.*, **6**, 109.

Weisburger, E. K., Weisburger, J. H. and Morris, H. P.—(1953) *Arch. Biochem. Biophys.*, **43**, 474.

Wiest, W. G. and Heidelburger, C.—(1953) *Cancer Res.*, **13**, 255.